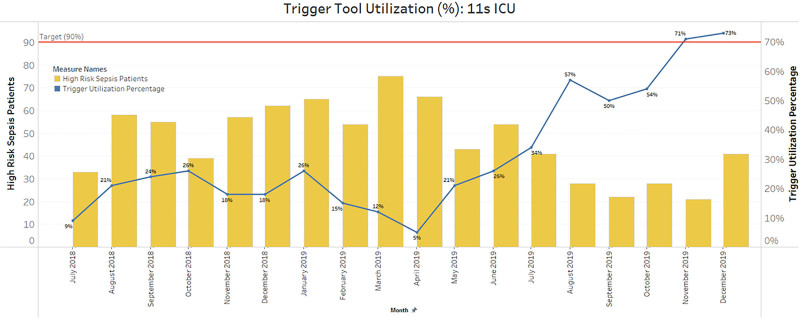# Increased Sepsis Screening Compliance in a Medical ICU Utilizing Rapid Process Improvement

**DOI:** 10.1097/pq9.0000000000000528

**Published:** 2021-12-16

**Authors:** Pascale Audain, Julie Vincuilla, Himi Mathur, Jenny Chan Yuen, Daniel Kelly

**Affiliations:** From the * Boston Children’s Hospital, Boston, Mass.; † Harvard Medical School, Boston, Mass.

## Abstract

The Children’s Hospital Association’s Improving Pediatric Sepsis Outcomes (IPSO) collaborative is a multi-center quality improvement (QI) learning collaborative of 61 U.S. children’s hospitals that seeks to improve sepsis outcomes through collaborative learning and reliable implementation of evidence-based interventions in pediatric emergency departments, intensive care units, general care units, and hematology/oncology units. Specifically, IPSO’s goals are to decrease sepsis-attributable mortality and prevent hospital-onset sepsis among children.

The following 10 abstracts represent a select group of projects undertaken by IPSO participating hospitals that were presented at one of three collaborative events in 2020 and 2021. IPSO’s Research Workgroup reviewed all submitted abstracts and selected the top 10 for inclusion in this Supplement

## Background/Significance:

Standardized assessment of sepsis risk and expedited treatment improves patient survival. The Medical ICU at Boston Children’s Hospital implemented a sepsis bundle, including a sepsis trigger tool, huddle, and orderset as part of the Children’s Hospital Association Improving Pediatric Sepsis Outcomes collaborative. The Sepsis Trigger Tool is composed of brief questions within the electronic medical record to identify the need for a Sepsis Huddle. If indicated, the patient’s medical and nursing team huddles at the bedside to establish a plan of care. Sepsis Trigger Tools are indicated for patients with new fever/hypothermia (temperature ≥38.5˚C or <36˚C) or new infection concern. Sepsis Trigger Tool compliance MICU was 21% at the launch of our participation in the Children’s Hospital Association Sepsis Initiative in August 2018.

## Purpose and Goals:

The aim of the study was to improve Sepsis Trigger Tool utilization in patients at risk for sepsis to facilitate early sepsis recognition and timely treatment with the goal to reduce mortality and improve patient outcomes.

## Methods:

Serial PDSA cycles were conducted from July 2019 through December 2019. Interventions were developed for identified barriers, and included: staff education, repositioning Sepsis Trigger Tool within the electronic medical record to improve accessibility and documentation compliance, daily auditing of cases to ensure appropriate reporting, and the development and implementation of daily staff surveys.

## Findings:

Serial PDSA cycles increased Trigger Tool utilization from 34% to 73%. Staff survey results identified improvement opportunities: staffs unaware that patients met improving pediatric sepsis outcomes sepsis criteria (30%), awareness of need for repeat evaluation if negative screen within 24 h (19%), and appropriate use of the improving pediatric sepsis outcomes tools but not properly documented (15%).

## Implications/Next Steps:

Continued identification of utilization barriers and direct feedback to staff is ongoing to improve sepsis risk surveillance.

**Figure:**